# Teaching empathy and compassion to healthcare providers in palliative care: a scoping review

**DOI:** 10.3332/ecancer.2024.1817

**Published:** 2024-12-12

**Authors:** Seema Rajesh Rao, Mithili Narayan Sherigar, Michelle Normen, Udita Joshi

**Affiliations:** 1 BHT-Karunashraya Institute for Palliative Care Educ ation and Research, 40 Varthur Road, Marathahalli, Bengaluru 560037, Karnataka, India; 2 School of Medicine, Cardiff University, Cardiff’dCF14 4YS, UK; 3 BHT-Karunashraya, 40 Varthur Road, Marathahalli, Bengaluru 560037, Karnataka, India; a https://orcid.org/0000-0001-8278-3640; b https://orcid.org/0000-0001-9148-890X; c https://orcid.org/0000-0002-9039-7894

**Keywords:** compassionate care, teaching, cancer, palliative care, healthcare professionals, experiential teaching, reflection

## Abstract

Empathy and compassion are core competencies that healthcare providers (HCPs) require when caring for patients and families with life-threatening illnesses like cancer. These constructs are often challenging to define and generalise and are often used interchangeably. Medical education has evolved from the traditional curriculum-based approach to a more eclectic competency-based approach. The purpose of this review is to explore the current evidence on teaching compassionate care for palliative care issues in cancer settings in lower-middle-income countries. A preliminary search of the Scopus database from 2,000 until now identified 1,502 records, of which 54 peer-reviewed articles were included in this review. Training in compassion and empathy was delivered in three formats: online, face-to-face and blended learning or hybrid. The training modalities were didactic, experiential and reflective, with many educational interventions using a multimodal approach. The educational interventions reported a positive outcome and improvement in empathetic and compassionate behaviours. However, they were limited due to inadequately defined constructs, use of self-reported outcome measures and difficulty in ascertaining if these skills were retained long-term and were translated into the clinical settings. Given that compassion and empathy are multidimensional constructs, it is imperative that educational interventions be multimodal and learner-centred, focusing on developing the knowledge, attitudes, skills and behaviours of the HCP in providing compassionate care while aiming for conceptual clarity regarding definition and more robust validated outcome measures.

## Introduction

As per GLOBOCAN 2020, approximately 19.3 million new cancer cases were reported globally, with 10.0 million dying due to cancer [1]. The lower- and middle-income countries (LMIC) share a disproportionately higher burden of cancer mortality, with access to and delivery of oncological services being suboptimal [2]. The cancer trajectory is punctuated by disability, distress and, in some cases, death, causing suffering to the patients, families and healthcare providers (HCPs) [3]. This suffering is described as an all-encompassing, pervasive, multidimensional phenomenon, often called ‘total pain’ [4]. Alleviation of suffering is the primary goal of medicine and is the foundation of the discipline of palliative medicine [5].

The comprehensive clinical model of suffering posits that suffering from an illness permeates every aspect of an individual’s life, including the biomedical, psycho-emotional, sociocultural and existential domains [6]. Compassion is an innate response to suffering and an essential aspect of high-quality healthcare [7]. Compassionate care is linked to positive outcomes for patients and their families, HCPs and the healthcare systems [8]. It improves the provider–patient relationship, decreases staff stress and burnout, and improves provider satisfaction [8, 9]. Patients who receive compassionate care feel respected and valued, experience more autonomy, are less anxious, report lesser pain and other symptoms, and exhibit better psychological adjustment to the cancer diagnosis [8, 9]. There is reduced caregiver burnout, shorter hospital stays and lower hospital costs [8, 9]. On the other hand, the absence of compassion can result in patient-provider conflicts, poor quality of life for patients and families, increase the risk of adverse medical events, and negatively impact HCPs public image, with detrimental legal and economic consequences [8].

Despite being an essential component of quality healthcare, studies have shown that HCPs within hospital systems find it challenging to address the multidimensional nature of suffering [6, 8–10]. The emphasis on the biomedical approach results in a decline in empathy among medical students and residents during the course of medical school [9, 11]. This is attributed to the medical socialisation process, wherein medical trainees are taught to focus primarily on the technical and diagnostic aspects of medical care and encouraged to be emotionally detached and neutral as they dehumanise and objectify the patient’s suffering [9]. When exposed repeatedly to patient and family distress, medical trainees use avoidance to cope with the situation and tend to withdraw physically and emotionally [12]. In addition, a lack of adequate modelling and training in empathetic and compassionate behaviours, inadequate mentoring, and a curriculum that undervalues human aspects of medicine all contribute to the erosion of empathy [8].

Given the value of compassion in healthcare and the compelling evidence of its benefits, recent recommendations emphasise the need to consider compassion as a core competency for HCPs, train and evaluate HCPs for compassion, and adopt and implement standards for compassionate care. There is an emerging need to restructure medical education, which focuses equally on developing evidence-based biomedical knowledge and skills and value-based humanistic skills of empathy and compassion [13]. However, there are significant lacunae in the academic literature about teaching compassionate care and its effectiveness. In this review, the current landscape of teaching compassion and empathy for healthcare professionals for palliative care issues is explored.

### Empathy, compassion and compassionate care

Compassion and empathy are key aspects of healthcare yet remain veiled in ambiguity, with numerous definitions and conceptualisations [14]. In academic literature, these two concepts are often used interchangeably, confusing one with the other [15]. However, recent papers have attempted to delineate them as separate but closely related concepts [16].

Empathy is defined as the ability to experience another’s emotion vicariously, that is, the ability to walk in another’s shoes [17]. Empathy is categorised into affective empathy, an ability to experience another’s feelings or emotions; cognitive empathy, an ability to understand feelings and perspectives objectively; and behavioural empathy, an ability to communicate this understanding to the patient [15]. Empathy is considered to be a precursor to compassion [18]. Compassion is a complex, dynamic, individualised and multifaceted construct [13]. From an HCP perspective, compassion is defined as a ‘virtuous and deliberate response to know a person, discern their needs and alleviate their suffering through relational understanding and action [14]. Compassion involves two processes; the first is an empathetic response to the patient’s suffering, and the second is a motivation to reduce the suffering followed by an accompanying action [15, 21]. Some authors reiterate that professional compassion is a conscious effort and includes the act of warning patients about future mortality and morbidity [8]. From the patient’s perspective, compassion, in addition, includes clinical competence [19]. Patients expect HCPs to provide effective evidence-based clinical care while connecting with them and their families meaningfully [19, 20]. For South Asian patients, compassion includes cultural competency, with HCPs exhibiting a non-judgemental attitude and accepting their beliefs and preferences [8]. Compassion in healthcare is conceptualised as a feeling, attitude, virtue and duty [15].

Compassionate care is defined as care that addresses the physical, emotional, social and spiritual pain and suffering of patients and their families, akin to ‘total care’ for ‘total pain’ [15, 20, 21]. Compassionate care incorporates a cognitive component, the ability to recognise, acknowledge and understand the patient experience; an affective component, the ability to imagine and feel what the patient is going through and the ability to regulate one’s own emotions; an altruistic component, the motivation to respond to the patient’s need selflessly; and an action component, choosing to act to alleviate the patient’s suffering by co-creating an action plan along with the patient [20, 21]. From the perspectives of palliative care patients, compassionate care is about the connection they experience with the HCP, the positive presence and warmth the HCP brings to the encounter, and about being treated with respect and dignity as a whole person rather than just a disease [22]. Thus, the HCP’s attitude, demeanour, body language, sensitivity and concern for the patient, authenticity, positivity, cultural sensitivity and medical competency influence the delivery of compassionate care [23]. Compassionate care thus becomes an integral component in healthcare, especially for patients nearing the end of life (EOL) and their families, and an imperative duty of every physician.

### Teaching compassionate care for palliative care issues – current evidence

Medical education has evolved over the decades, shifting from a conventional content-based approach to a more eclectic competency-based training [24]. Compassion is one of the core competencies in healthcare, with patients and caregivers considering compassionate care an important indicator of quality healthcare [25]. The emergence of the biopsychosocial approach to health and emphasis on whole-person care has intensified the efforts to embed training in compassion into the healthcare curriculum [26]. Recent evidence suggests that the qualities of empathy and compassion can be nurtured in HCPs, and compassionate care behaviours taught through formal healthcare education [8]. However, with compassion having different connotations in different settings, defining and quantifying the term becomes challenging when developing training programs. In addition, across healthcare literature, the terms empathy, compassion, caring and compassionate care are often conflated, creating conceptual ambiguity [8, 26]. Further, compassion is facilitated by the inherent personality traits, attitudes and emotions of the HCP, their life experiences and their worldviews [8]. While there is ample evidence about the need to teach empathy and compassion to HCPs, there is also uncertainty about the components of this training and its operationalisation in healthcare settings. While medical educators agree unanimously that conventional teaching methods may not be effective for teaching compassion, there is a lack of consensus about effective teaching methodology [8].

## Methods

### Review question

What is known from the literature about teaching empathy/compassion to HCPs for palliative care issues?

### Review design

In this review, the authors intended to explore the current evidence on teaching compassion and empathy to healthcare professionals for palliative care issues in LMIC. A preliminary search of the Scopus database combining the search terms ‘teaching’ AND ‘compassion’ AND ‘palliative care’ AND ‘HCPs’ AND ‘LMIC’ was conducted. Given the interconnectedness of the terms compassion and empathy, we kept the search terms broad to include both terminologies. Given the paucity of literature generated by this search, the authors decided to reframe the research question by omitting the term LMICs

A scoping review methodology was chosen as the research question is exploratory in nature, and this study aimed to use a systematic approach to map a broad range of literature in this area and help identify knowledge gaps. The review followed Arskey O’Malley’s framework [85], with Levac, Colquhoun and O’Brien enhancements which include the following [84]: i) identifying the research question, ii) identifying relevant studies, iii) study selection, iv) charting the data and v) collating the results. The methodological quality of the included studies is not formally assessed in this.

### Search strategy

Two reviewers searched the electronic databases, including MEDLINE (via OVID), APA PsycINFO (via OVID), CINAHL (via EBSCO) and the multidisciplinary database Scopus. Full search strings for each database are available in Appendix 1. The reference list of included studies was searched to identify additional relevant studies.

### Inclusion and exclusion criteria

Articles published in indexed journals in English from 2000 to 2023 were included in the review For the purpose of this review, only articles that focused on educational programs, curricula or interventions to enhance compassion or empathy for HCPs (medical, nursing, dental, pharmacy) in the context of palliative care were included. We excluded studies focusing on other related concepts like communication skills, compassion fatigue, self-compassion, ethics, mindfulness and the neurobiology of compassion and empathy. Commentaries, letters, conference abstracts and abstracts for which full-text articles could not be retrieved were excluded from the review.

### Data extraction and analysis

All identified articles were exported to a reference manager software EndNote 21, and duplicates were removed. The team members (UJ, MS) screened titles and abstracts to identify studies that met the inclusion criteria in the online systematic review software Ryaan. Full-text articles were retrieved, and any difficulties with inclusion were resolved through discussion with co-authors (SRR, MN), who assessed the eligibility of the study independently. Any disagreements were resolved by consensus through meetings and discussions.

Data was extracted and charted in a standardised template. The data extraction sheet included: a) article information (title, author, year of publication and journal name), b) setting information (country), c) research information (aim, design, sample size, population), d) definition of empathy and compassion, e) teaching interventions (modalities of teaching, effectiveness, facilitators and barriers). We summarised the data across studies using a content analysis approach. We did not critically appraise the included studies for quality because of the methodological heterogenicity and the scoping review methodology.

We were able to identify 1,502 records. The titles and abstracts of 1,502 records were screened for relevance, and 90 full-text articles that could be potentially relevant to the study were retrieved. Of these, 54 articles [27–80] were included in this review. Given the need to understand the existing landscape of teaching compassion and empathy for palliative care issues in healthcare settings, a mix of qualitative studies, quantitative studies, mixed methods, reviews and anecdotal evidence are included in this review.

### Concept of empathy and compassion – current evidence

The common theme across all studies is the recognition of empathy and compassion as a core competency of quality healthcare while emphasising the need to revamp healthcare education by focusing on the humanistic aspects of care [27–79]. The studies also reiterate the decline in empathy and compassion during medical and nursing education, attributing it to the lopsided focus on biomedical education [27–77]. Some studies have delineated empathy and compassion as two separate constructs, with some studies using the constructs interchangeably while discussing educational interventions [27–76]. One study has conflated the two terms [50]. Approximately 50% of the studies did not define either of the constructs [32–35, 37, 39, 40, 42–44, 46, 48, 49, 58, 60, 61–64, 66, 68, 69, 71, 75–80]. Compassionate care and its process components are not defined in the studies. However, the elements of compassionate care enumerated in the various studies include effective and affective communication skills, patient-centred care, professionalism, goals of care discussion, shared-decision making, managing spiritual and existential issues, perspective-taking, self-awareness, professionalism, teamwork and interprofessional collaborations [27–80].

**• Population and settings**

The majority of studies highlight compassion and empathy as an essential component of all HCP-patient interactions, especially in situations where suffering is inherent, like chronic terminal illnesses and at EOL. However, it is evident in the studies that healthcare professionals and students are unprepared and uncomfortable while dealing with these issues [27, 28, 31–35, 36, 37, 39, 43, 45, 51, 52, 54, 56, 60–65, 70–78]. While empathy and compassion are considered innate dispositions, there is evidence to suggest that they are teachable skills [27, 28, 38, 42, 47, 50, 51, 52, 55]. However, opportunities for such training in healthcare education are fragmented, occurring *in silos*, with inadequate curriculum and ineffective format [62]. To offset this, some studies suggest incorporating components of training in empathy and compassion during the preclinical or early years of healthcare education [27, 28, 31–35, 36, 37, 39, 43, 45, 51, 54, 55, 56, 60–65, 70–78]. Given the evidence regarding the decline in empathy during clinical training, some studies suggest that it may be more effective to embed it in the curriculum across all years of healthcare education [62].

Medical students undergo training to enhance compassion and empathy either during their undergraduate training during medical school [31, 33–36, 48, 50, 54, 55, 62–64, 66, 68, 78, 79], incorporated into internal medicine, family medicine, psychiatry, oncology, or neurology clerkships or as electives [79] or during residency [31, 33–35, 65, 68, 78]. Some medical professionals (palliative care providers, physicians and oncology professionals) undergo the training as part of their staff development program [28, 32, 46, 47, 58]. Undergraduate nursing students [39, 40–42, 44, 45, 52, 77], pharmacy students [49, 59], senior nurses [37, 38, 43, 56, 80], psychologists, physical therapists, occupational therapists, speech-language therapists, social workers, chaplains, hospital administrators, drama students and medical educators [29, 30, 51, 57, 60, 61] undergo training in compassion and empathy which is embedded in palliative care/end-of-life care (EOLC) or oncology modules. Compassion/Empathy is the primary focus of the curriculum in one study [41] in this review. In the majority of the studies, it is subsumed under other existing topics like oncology, palliative or EOL care and interprofessional education.

### Interventions for enhancing compassion and empathy for palliative care issues

**• Modalities of teaching compassion and empathy**

The training programs for compassion and empathy are delivered online, face-to-face in classroom or clinical settings or in a blended learning or hybrid format [27–80].

### Online format

Most studies have utilised in-person experiential instructional methods to teach empathy and compassion. In-person training is expensive and is limited in terms of accessibility and flexibility. Online training aims to overcome this limitation by delivering more flexible, easily accessible programs which facilitate self-regulated learning.

The e-learning modules [30, 41, 61] covered specific aspects of EOLC, namely patient-centred care, shared decision-making, compassionate care, communication and goals of care. Two online modules, the End of Life Essential Modules [30] and the Emergency Department EOLC Module [61], utilised a case-based learning approach, which aided the application of learned knowledge into real-life scenarios. Compassion and empathy were embedded in the EOL module, with compassionate care being one of the competencies. Pre- and post-evaluation of the module data and free text responses from participants suggest a positive impact on the knowledge, skills and attitudes of the learners and increased confidence in providing EOLC.

The online compassion module for nurses [41] incorporated reflective assignments and discussions with tutors and peers to make the learning interactive. Qualitative end-of-module assessments indicate that learners, in addition to acquiring knowledge, also developed insight into their attitudes, values and beliefs and described compassionate behaviours they would adopt in the clinical setting towards patients, colleagues and themselves. An online lecture on Medical Comics in Palliative Care [48] used comics with graphic illustrations to deliver content on skills for compassionate palliative care highlighting the stories of patients, caregivers and HCPs. Most medical students found these skills essential and the teaching method engaging.

### Blended or hybrid format

The blended programs include an interprofessional simulation education program for nursing students, the Team Strategies and Tools to Enhance Performance and Patient Safety (STEPPS) Program [42], and a 4-year integrated curriculum on EOLC for medical students [62]. Both programs incorporated blended learning elements with online didactic modules, interactive discussions, experiential skill-building activities, simulated interprofessional team meetings and standardised patient-caregiver assessments.

Empathy is one of the primary outcomes of the STEPPS program, which was evaluated through a post-seminar survey. Nursing students posit that the classroom lectures introduced them to the concept of empathy (knowledge), while clinical rotations provided them with an opportunity to observe empathetic and non-empathetic behaviours in mentors and peers along with an opportunity to apply the learned knowledge (skill). Prior personal and professional experience influenced their empathetic ability (attitude), while the simulation in the Team STEPPS program provided an opportunity to practice the behavioural skill in a non-threatening space [42].

Elements of compassionate care are embedded in the 4-year longitudinal EOLC curriculum. Students progress through case-based EOL discussions, simulations, hospice visits and communication workshops, followed by self-reflection activities and discussions with mentors. The program was evaluated through student reflections, surveys from graduating students and observed structured clinical examination. Medical students demonstrated basic competencies in symptom management, communication and spiritual/cultural and interprofessional elements of care. The emerging themes from the student reflections indicate empathy, an understanding of the patient and caregiver experience, the impact of the compassionate presence of the HCP, and death acceptance [62].

### Face-to-face format

In the face-to-face format, educational interventions for compassion and empathy are offered either in the classroom or clinical settings and utilise multiple modalities for teaching. Didactic lectures on pain, symptom management, communication, ethics and EOL are combined with other experiential teaching modalities. To enhance learning, these didactic lectures are followed by interactive teaching methods like in-class debates, clinical vignettes, case discussions and reflections. Changes in attitudes towards death and dying and a better understanding of the construct of empathy were reported by the learners when multiple teaching modalities were combined [31, 32, 34–37, 39, 40, 43–46, 49–61, 63–66, 70, 71, 74, 76–79].

Experiential teaching techniques include clinical rotations in palliative care and oncology and hospice or visits to hospital palliative care units [27, 31, 34–36, 43, 54, 57, 64, 68, 69, 78]. The learners were required to submit written reflections after these visits, which were evaluated. Themes of compassion and empathy were evident in these reflections, with learners identifying with patient and family perspectives, respecting personhood beyond the disease, discovering the humane side of medicine and the nuances of interprofessional teamwork. Rotating through palliative care and oncology was a transformative and meaningful experience for these learners [27, 31, 34–36, 43, 54, 57, 64, 69, 78]. When learners ascribed meaning to their experience with terminally ill patients, they exhibited a high sense of empathy [68]. One study hypothises that if physician trainees are taught to find meaning in their experience with the patients, it can lead to higher empathy levels [68]. Passive learning through observation of palliative care physicians and nurses and role modelling also contribute to enhanced empathy [27, 31, 35, 54, 53].

Other experiential activities include learners completing their advance directives [33, 34, 49], discussing mortality in the death over dinner [49, 76] and Wit Educational initiative, where learners watched the drama/movie Wit, followed by reflection and discussion [55, 59, 65, 78, 79], and a digital story-telling training program [80]. These were coupled with didactic sessions on EOL and palliative care. Written reflections, pre- and post-surveys and semi-structured interviews were the method of evaluation and indicated improvement in knowledge, skills and attitude in EOLC. Learners demonstrate improved empathy and compassion, which was subsumed under the competencies for EOLC. Active teaching strategies like debates and small and large group discussions were incorporated into EOL, death and dying and spiritual modules and coupled with other teaching modalities [34, 66, 59].

Problem-based teaching through patient storytelling and patient interviews [34, 36, 54, 69, 71, 74], immersive service-learning experiences [37, 43, 70], role plays [32, 44, 54, 66, 71] and game-based learning [50] have also been utilised for teaching compassion and empathy to medical and nursing students.

The Comfort Shawl Project is an immersive service-learning project wherein nursing students undergo palliative care rotation with a hospital-based unit. During this time, they gift handcrafted shawls to patients allowing for informal interaction. Participant reflection indicates that this learning experience goes beyond nursing care to focus on compassionate communication and support [37, 43]. Immersion through volunteering enhances empathy and compassion and is protective against empathy decline seen during healthcare education [70].

Learner-centred role plays integrated into communication skills workshops [32], EOL workshops [44, 71], palliative care training [54] and undergraduate spiritual care courses [66] allow learners to step into the shoes of the patient and their families and are effective experiential learning techniques. Evaluation through reflective writing and pre- and post-workshop questionnaires indicate enhanced empathy and communication skills, in addition to increased basic palliative care competencies. The Empathy Project – A Skill Development Game is a novel tabletop game for small groups aimed at building empathy in learners [50]. The learners write empathic responses to challenges outlined in the card game. The facilitator and peers review the responses, provide individual feedback and guide the learner towards making empathetic statements. An unvalidated measurement tool developed for this game was used to evaluate outcomes. This tool indicates increased use of empathetic language in learners who played the game. Unlike other teaching strategies, this game-based skill development game had empathy as the primary outcome.

A number of studies have used simulation activities [39, 44, 46, 49, 56, 60, 71], symptom simulation [46], participatory drama and sociodrama [40, 45, 58] and Virtual Simulation Lab [53] to enhance compassion and empathy.

High-fidelity simulation of scripted EOL clinical situations focused on nursing assessment, technical skills of symptom management and medication administration, caring behaviours and interprofessional communication. Nursing students trained in a simulation room resembling a hospice or a hospital palliative care unit under the guidance of expert facilitators [39, 44, 46, 49, 56, 60, 71]. The evaluation included debriefing after the simulation exercise, reflection, semi-structured interviews and simulation effectiveness tool -modified. The simulation training seeded the learners with the knowledge, skills, attitudes and behaviours required for caring for dying patients, being with the bereaved and dealing with colleagues. Learners reported improved confidence in managing EOL issues. They were able to communicate empathetically with patients and caregivers, understand and respect their views and feelings, demonstrate compassionate caring behaviours, exhibit better interprofessional communication and develop greater awareness about self-care. Some learners found the experience distressing, while the majority reported a positive experience with simulation, attributing it to the presence of experienced facilitators who provided them with a safe space for expressing themselves [39, 44, 46, 49, 56, 60, 71].

In the Symptom Simulation Lab, learners experience first-hand the debilitating effects of chronic illnesses like breathlessness, fluid retention, vision impairment, etc., in a 5-minute simulation session. The learners complete a pre- and post-simulation survey to assess empathy and undergo debriefing and reflection. The simulation session resulted in increased perceived empathy for patients and their caregivers. This increased level of empathy lasted even 10 months after the simulation training [46].

Drama Combined Nursing Education for Cancer Care [40], communication skills workshop for palliative and EOLC utilising participatory drama techniques [45], and sociodrama on difficult communications for faculty and staff in oncology are some of the interventions that cultivate compassion and empathy. A learner enacting the role of the patient, caregiver or colleague is able to understand and experience the feelings and emotions of the other person. Sociodrama techniques of doubling and role reversal help the learner articulate the hidden feelings behind the emotional reactions of the patient/caregiver. The practice sessions provide an opportunity for the learners to reinforce verbal and nonverbal empathetic skills that could be translated into their practice every day. Evaluation based on debriefing, reflection and semi-structured interviews demonstrated a significant increase in the learner’s understanding and empathy for patients, caregivers and colleagues.

Virtual reality has been used to train HCPs in compassion and empathy. First-year medical students experienced the journey of a patient, from receiving the diagnosis of cancer to transitioning into home hospice care, through the Embodied Labs module. Learners were able to immerse themselves in the journey of the patient first-hand and experience the feelings and emotions of the patient, the caregivers and the HCPs. Data collected through pre- and post-survey indicate high levels of immersion and embodiment. Embodiment positively correlated with empathy. Learners reported vicariously experiencing the emotions, feelings and physical symptoms of the patient. In addition, learners reported increased confidence in managing EOL issues.

### Evaluation of outcomes

There was no uniform method of evaluating the training outcomes. Outcomes were evaluated both qualitatively and quantitatively through surveys, debriefing, reflective techniques, evaluating the apprenticeship journals and pre and post-evaluation or using validated scales like the Response Empathy Scale [66] and the 30-item Balanced Emotional Empathy Scale [59], Frommelt Attitudes Towards Care of the Dying, Compassion Competency Scale, or interpersonal reactivity index [34, 35, 44, 49, 51, 54, 57, 59, 63, 64, 66, 68, 71, 77, 80].

## Discussion

This review explored the current literature on teaching compassion and empathy in the context of palliative care to HCPs.

What is evident in this review is that there is a lack of consensus on how the constructs of compassion and empathy were defined, observed, expressed and measured. This is consistent with previous literature. The studies in this review also emphasise that compassion and empathy are not just innate traits but teachable skills that drive prosocial behaviour [81, 13]. The studies also underline that early exposure of HCPs to elements of death, dying and bereavement during undergraduate training has the potential to foster compassion and empathy. However, there was no consensus as to when the training should be initiated, the duration of the training, what should be included in the curricula and how the effectiveness of the training should be measured. As is evident above, many of the interventions were heterogeneous, and given this ambiguity, conceptualising a framework and curricula for teaching compassion/empathy becomes challenging.

Despite the heterogenicity, the dominant understanding across all studies was that the traditional curriculum-based healthcare education model does not prepare HCPs to care for someone at EOL. The conventional didactic approach is teacher-centric and content-rich, focusing on the knowledge domain with an emphasis on memorisation and recollection [35]. In teaching compassionate care, this model would be ineffective as the compassionate care process involves an interplay of knowledge, attitudes, skills and behaviours that facilitate a transformative change in the learners [27, 82]. A competency-based model that focuses on attitude, skills and behaviour, in addition to knowledge, is ideal for teaching compassion and empathy. As seen in the review, a wide range of teaching pedagogies has been utilised for cultivating compassion and empathy in the context of palliative care.

Given that most of the training programs used to teach empathy and compassion adopted the experiential learning pedagogy, we interpreted the findings of the review based on Kolb’s Experiential Learning Theory (ELT). ELT posits that the learning cycle traverses through four stages: a) concrete experience (feeling), b) reflective observation (observing), c) abstract conceptualisation (thinking) and d) active experimentation (doing) [83]. Effective learning occurs when the learner progresses through the four cycles.

The hospice visits, rotations in palliative care, virtual and physical simulation labs, role plays, service learning and watching a drama or movie (WIT) provided the learners with a concrete EOLC experience. These immersive EOLC experiences provided learners with the opportunity to observe both the suffering of the patients and their families as well as the compassionate behaviours of the palliative care experts, peers and other senior team members. However, whether these behaviours were adopted or not depended on whether these encounters were perceived as rewarding or distressing. Studies show that HCPs find managing terminally ill patients distressing. Supervised clinical encounters and expert mentoring enhance coping and help the participants adopt new behaviours that convey empathy and compassion [83].

The second and third stage of ELT is reflective observation and abstract conceptualisation. As seen in this review, reflection as a pedagogical tool was widely used in many empathy and compassion training activities. The process of reflection helped participants analyse their experiences from different perspectives and helped them identify and understand their own reactions to the situation and that of others. Abstract conceptualisation is the culmination of reflective observation leading to new learning that facilitates a shift in attitude. The learners tried to make sense of what they saw and felt during the immersive EOL experiences. Many of the training programs in this review encouraged learners to transform their experiences into something meaningful by having post-intervention debriefing, group discussions, debates and journaling, which followed the immersive experiences.

The final stage of the ELT is active experimentation, wherein the learners test the new learning in the real world [83]. The role plays, simulation labs and clinical rotations helped learners test their newly acquired skills in simulated real-world situations. However, most of the training programs in this review were cross-sectional, provided over one point in time, varying between a few hours to a few months, making it difficult to ascertain whether any long-term behavioural and attitudinal change was achieved.

Though most studies reported positive outcomes, they are limited due to the study design and outcome measures used. Only two studies [59, 66] used validated scales to measure empathy. The majority of the studies used self-report outcome measures. As a result, it is difficult to ascertain if any behavioural change occurred, if it was retained long-term, or if it was integrated into clinical practice. Only one study explored the long-term effects of training [46].

Some studies indicate that online modules and blended learning programs that incorporated elements of experiential learning were also successful in enhancing empathy and compassion among HCPs. The use of multimodal teaching techniques delivered through blended learning would enhance the reach and scope of the training in compassion and empathy.

This review highlights the knowledge gaps in training healthcare workers in compassion and empathy. Many of the studies are from high-income countries (US, UK, Australia, Canada, New Zealand, Israel, Slovenia, South Korea, Austria, Italy, Ireland, Taiwan ) or upper-middle-income countries (Turkey, Ecuador) wherein heterogeneous training programs have been incorporated into healthcare curricula. Longitudinal training programs, experiential learning pedagogy with supervised encounters, and early initiation of training for HCPs might be beneficial in facilitating empathy and compassion. However, the application of many of these programs, like virtual and physical simulation labs, may not be feasible in low-resource settings. Given the low penetration of palliative care in LMICs, rotation in palliative and EOLC may not be a feasible option for experiential learning.

## Strengths and limitations

This review has attempted to map the available literature on how compassion and empathy are taught to HCPs for palliative care issues. The limitation of this study is that we have not consulted with a librarian for the search strategy due to logistic issues, and have not registered the protocol.

## Conclusion

This review enumerates various educational interventions developed to cultivate empathy and compassion in HCPs in palliative and EOLC. Most educational interventions used multimodal teaching pedagogies, dominated by experiential teaching techniques. However, the true potential of these interventions remains unexplored in view of the methodological limitations. Given that compassion and empathy are multidimensional constructs, it is vital to incorporate techniques that enhance knowledge, attitudes, skills and behaviours into the training program and have a clear evidence-based definition of these concepts, with validated outcome measures for evaluation.

## Conflicts of interest

The author(s) declare(s) that there is no competing interest or conflict of interest.

## Funding

This review did not receive any specific grant from funding agencies in the public, commercial, or not-for-profit sectors.

## Author contributions

All authors have contributed towards design, conduct, analysis and manuscript writing.

## Figures and Tables

**Figure:**
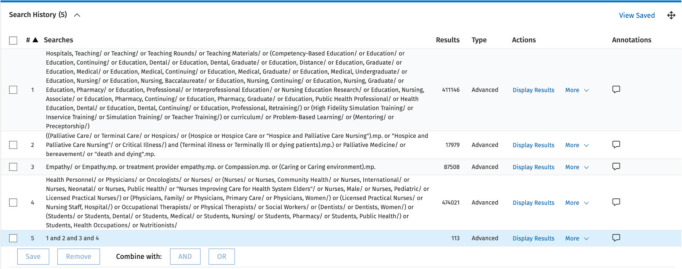
MEDLINE search strategy.

**Figure:**
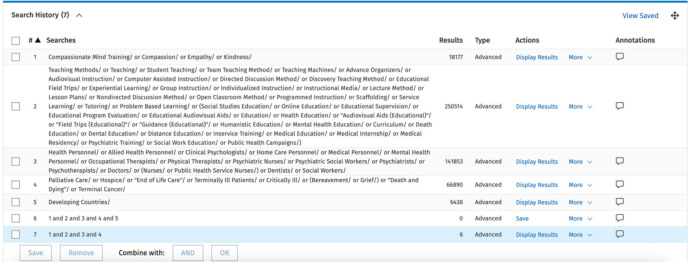
PsycINFO search strategy

**Figure:**
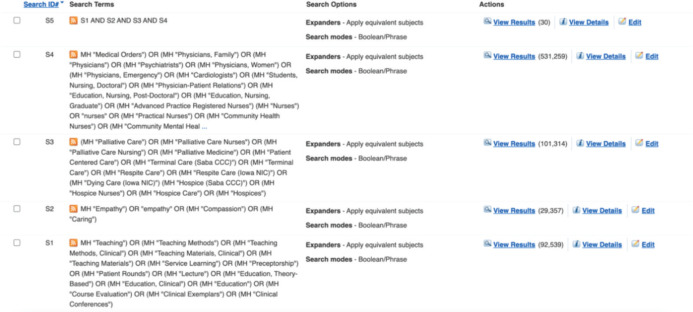
CINAHL search strategy

**Figure:**
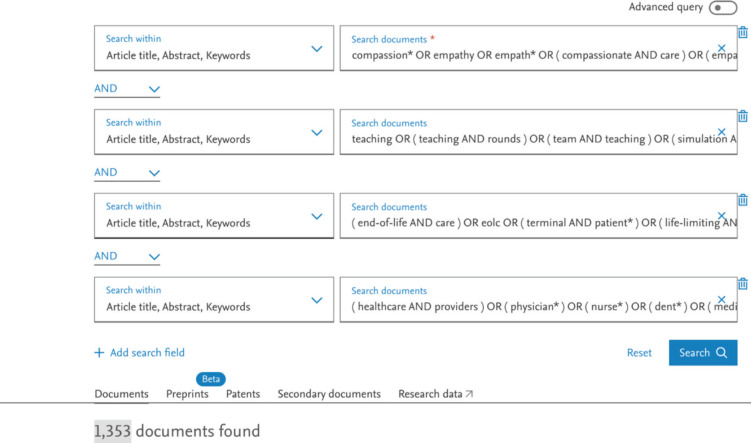
SCOPUS search strategy
